# Morphological changes in bitches endometrium affected by cystic endometrial hyperplasia - pyometra complex – the value of histopathological examination

**DOI:** 10.1186/s12917-021-02875-0

**Published:** 2021-04-26

**Authors:** Magdalena Woźna-Wysocka, Marta Rybska, Beata Błaszak, Bartłomiej M. Jaśkowski, Magdalena Kulus, Jędrzej M. Jaśkowski

**Affiliations:** 1 Department of Medical Biotechnology, Institute of Bioorganic Chemistry, Polish Academy of Sciences, 12/14 Z. Noskowskiego St, 61-704 Poznań, Poland; 2 Department of Preclinical Sciences and Infectious Diseases, Faculty of Veterinary Medicine and Animal Sciences, Poznan University of Life Sciences, Poznań, Poland; 3 Department of Tumour Pathology, Grater Poland Cancer Centre, Poznań, Poland; 4 Department of Reproduction and Clinic of Farm Animals, Faculty of Veterinary Medicine, Wroclaw University of Environmental and Life Sciences, Wrocław, Poland; 5 Department of Veterinary Surgery, Institute of Veterinary Medicine, Nicolaus Copernicus University in Torun, Toruń, Poland; 6 Department of Diagnostics and Clinical Sciences, Institute of Veterinary Medicine, Nicolaus Copernicus University in Torun, Toruń, Poland

**Keywords:** Uterine disease, Bitch, CEH, Pyometra

## Abstract

**Background:**

Cystic endometrial hyperplasia-pyometra complex (CEH-P) is one of the most common uteropathies in bitches. In diseases with mild or obscure clinical signs and normal uterine size, a diagnosis based on a clinical assessment might be incorrect.

The main aim of the research was to determine the morphological variables accompanying uterine diseases in bitches in microscopic evaluation. Consequently, the obtained results can be used to create a new classification system for uterine pathological changes during the development of the CEH-P, diagnosed by microscopic examination in bitches. Material for the study consisted of the uteri of 120 female dogs, aged 1–16 years, obtained during routine ovariohysterectomies. Macroscopic observation after a longitudinal incision of the uterine horns, allowed a preliminary classification of the uteri into research groups: control group (physiological uteri), and groups GI-III uteri collected form bitches with varying degrees of endometrial pathology. These preliminary classifications were then verified by histological analysis (H&E stain).

**Results:**

The obtained results made it possible to determine and describe the prevalence (%) of pathological changes characteristic of the analyzed uterine diseases in the examined bitches. Histopathological analyses that were conducted have confirmed preliminary macroscopic evaluation for the control group, group GII (CEH), and group GIII (pyometra). In the uteri of the GI group, a severe congestion of the endometrium has been observed – this is typical of inflammation – which was not confirmed during histopathological examinations. However, these examinations revealed acute endometrial haemorrhage of varying severity.

**Conclusions:**

Early reproduction disorders in bitches are, in general, not confirmed by clinical signs in the examined animals. The results show that during classification of typical morphological changes in the endometrium over the development of the CEH-P complex in bitches microscopic examinations are required. The obtained results indicate a frequent lack of consistency in the macroscopic assessment and histological analysis of the endometrium, observed in the analyzed uterine diseases, which in most cases is not followed by clinical symptoms. The presented classification of uterine diseases may be useful as a diagnostic tool in reproductive disorders in bitches and in examination in the field of basic research.

## Background

Over the last few decades, a growing trend of problems associated with canine reproduction, which has been a major obstacle in the practice of breeding these animals, has been observed [1–4]. A syndrome in the literature known as *cystic endometrial hyperplasia - pyometra complex* (CEH-P), identified within the reproductive system of female dogs, is the most serious and most frequently diagnosed pathological condition of the uterus in this species. It occupies a leading position among uteropathies found in the elderly females [1, 5–7]. The available literature states that pyometra can coexist with cystic endometrial hyperplasia of the glands, although this is often not necessarily the case. Both conditions may be separate uteropathies and are often diagnosed independently [6, 8, 9]. Cystic endometrial hyperplasia of the glands is not always preceded by pyometra. The average age of bitches with CEH and pyometra is very close and that is why pyometra does not always appear in bitches older than those with CEH [9].

During recent years, very young individual cases have been frequently reported [4, 10, 11]. In spite of numerous studies conducted on determining the causes of the development of the most common uterine pathologies included in CEH-P, the etiopathogenesis of this disease remains unclear [5, 6, 9, 12–17]. In addition, the prevalence of the diagnosis of this type of uteropathies is significantly higher in bitches compared to females of other species. This points to the existence of specific mechanisms that lead to disturbances in the functioning of the endometrium characteristic for dogs [18, 19].

According to current knowledge, the origination of CEH-P in bitches is the result of the simultaneous influence of hormonal factors and infectious agents. Abnormal ovarian activity is, therefore, important for the development of degenerative processes of the endometrium [2, 8]. The pathogenesis of endometrial hyperplasia in relation to conditions such as endometritis, cystic endometrial hyperplasia glands (CEH), pyometra as well as the possibility of their progression to a life-threatening stage, has become the research topic of numerous scientific papers [1, 20–23].

In contrast to the classification used in humans, the lack of a unified system of differentiation of the early histological changes covering the mucous membrane of the uterus in bitches leaves many uncertainties in the specification and differentiation of particular disorders [24]. The observed changes in the endometrium accompanying CEH bitches in the present study would amount to the category of complex hyperplasia (CH).

The routine clinical examination commonly used in veterinary practice, consisting of ultrasound or blood examinations, is an insufficient method for early diagnosis of pathological conditions of the uterus [6, 17]. According to cited authors, females affected by diseases like CEH- mucometra do not show any visible clinical symptoms, while diseases such as CEH - pyometra are obvious disease conditions A diagnosis given solely on the basis of clinical symptoms in the case of pyometra may be very diverse and can lead to serious errors. Pyometra accompanied by other symptoms such as: insomnia, depression, loss of appetite, polyuria, thirst and vomiting, can easily be confused with kidney failure, liver disease, diabetes or adrenal insufficiency [7, 9, 25, 26].

Biopsy of the uterus is a recommended test, which is considered to be one of the most accurate procedures for providing information about the actual condition of the organ [20, 27]. Christiansen et al. [28], using a transcervical biopsy, demonstrated both its high sensitivity and accuracy in identifying CEH, *endometritis* as well as uterine fibrosis.

The importance of the diagnosis of infertility using surgical biopsy through laparotomy is also emphasized by Mir et al. [27]. However, while the use of transcervical uterine biopsy to evaluate the condition of the organ is not a problem in most species, in dogs it is relatively difficult and involves the risk of organ damage and the development of infection [1, 12, 27–29]. Therefore, this method is not often practiced in the diagnosis of uterus diseases and etiology of reduced fertility.

Both endometritis, CEH and CEH-P complex are pathological conditions affecting the uterus, which are substantially distinguishable from each other by histopathological examination. The main aim of the research was to determine the morphological variables accompanying uterine diseases in bitches (CEH-P complex and endometrial haemorrhage in uterus) in microscopic evaluation. Consequently, the obtained results may be useful in creating a new system of classification of uterine changes and/or helpful in preparation for the diagnosis of reproductive disorders in bitches.

## Results

### Control group

**Fig. 1:**
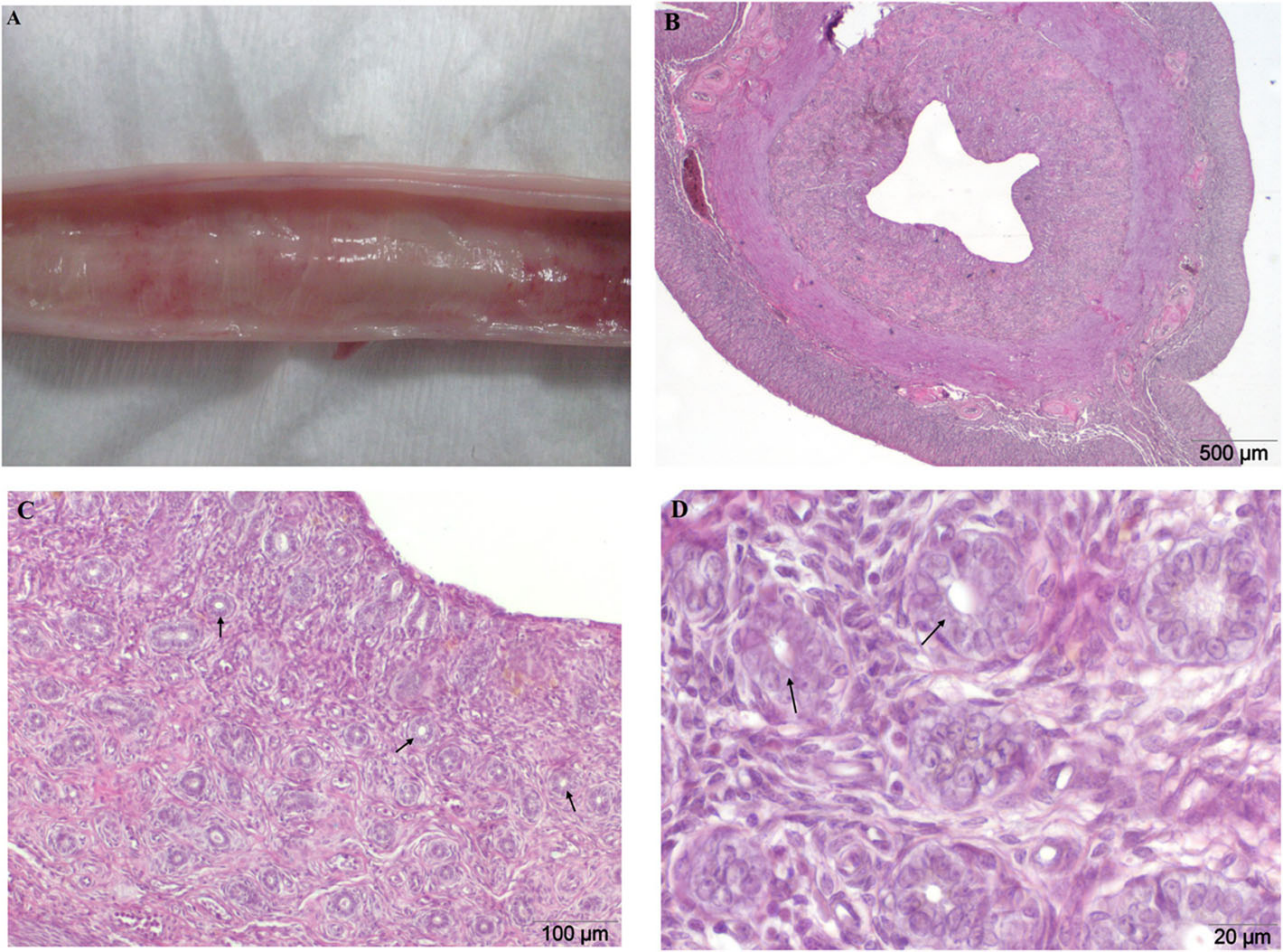
**a-d** Physiological uterus of bitch during *anestrus*. Physiological uterus of two-year-old bitch. **a** Macroscopic image, longitudinal incision of the uterine horns. No visible degenerative changes on the surface of the endometrium. **b**, **c**, **d** H&E stained histological images of transverse uterine horn fragment. A microscopic image of a typical physiological condition, without pathological changes in the structure of each layer of the uterus. Endometrial glands (arrows). Magnification: **b** 20x; **c** 100x, **d** 400x

Assessing microscopic preparations made from slices of normal uterine horns, no abnormalities in the morphology of its individual layers were found (Fig. 1b, c, d). The obtained microscopic image was typical of the expected stage of *anestrus* in the reproductive cycle of bitches on the basis of which the qualification of the uteri in the control group was made. Endometrium in this stage was regressed, covered with a layer of simple cubic surface epithelium. Crypt regions of the epithelium were hardly notable. The endometrial glands showed no secretory function and were atrophic. Reduced cellular cytoplasm of all uterine walls resulted in high nuclear density. Thus, approving the initial macroscopic evaluation, thereby showing no apparent pathological changes on the surface of the endometrium (Table 1).

**Table 1:** Prevalence n (%) of histopathological changes in full-thickness in the uterine horns obtained from 120 bitches

Group	Control	GI	GII	GIII
Number of bitches	30	30	30	30
Mean age of bitches (years) ± SD	2,2 ± 1,5^*^	4 ± 2,4^*^	6,4 ± 2^*^	9,3 ± 2^*^
Mean weight of bitches (kg) ± SD	17.1 ± 12,3	18.7 ± 11,3	20.2 ± 8,9	19.5 ± 14,8
**Variable of macroscopic evaluation**				
Significant uterine enlargement	–	–	12 (40)	30 (100)
Reddened of endometrium	–	30 (100)	9 (30)^a^	4 (13,3)^a^
Cysts on endometrium surface (full/ ruptured)	–	–	30 (100)	30 (100) 6 (20) 24 (80)
Discharge inside the horns:	–	–	3 (10)	30 (100)
-purulent	–	–	–	30 (100)
-serous-bloody	–	–	3 (10)	–
**Variable of histopathologic evaluation**				
Endometrial edema	–	24 (80)^A^	9 (30)^A^	–
-mild	–	–	7 (23,3)	–
-moderate		17 (56,7)	2 (6,7)	–
-severe		7 (23,3)	–	–
Acute endometrial haemorrhage:	–	30 (100)	9 (30)	4 (13,3)
-mild	–	6 (20)	3 (10)	–
-moderate	–	24 (80)	6 (20)	3 (10)
-severe	–	–	–	1 (3,3)
Cystic hyperplasia of endometrial gland	–	–	30 (100)	30 (100)
-mild	–	–	4 (13,3)	2 (6,7)
-moderate	–	–	11 (36,7)	9 (30)
-severe	–	–	15 (50)	19 (63,3)
Adenomyosis	–	–	2 (6,7)	4 (13,3)
-mild	–	–	1 (3,35)	–
-moderate	–	–	1 (3,35)	3 (10)
-severe	–	–	–	1 (3,3)
Endometritis	–	–	3 (10)	30 (100)
-chronic	–	–	2 (6,7)^a^	30 (100)^a^
-acute	–	–	1 (3,3)	–
Interstitial fibrosis	–	–	2 (6,7)	5 (16,7)

^*^ significant differences among control group and GI, GII, GIII, *P* value < 0,01; ^A^ significant differences between GI and GII, *P* value < 0,01; ^a^ significant differences between GII and GIII, *P* value < 0,05; n-number of animals with changes in the uterine

### Experimental group I

**Fig. 2:**
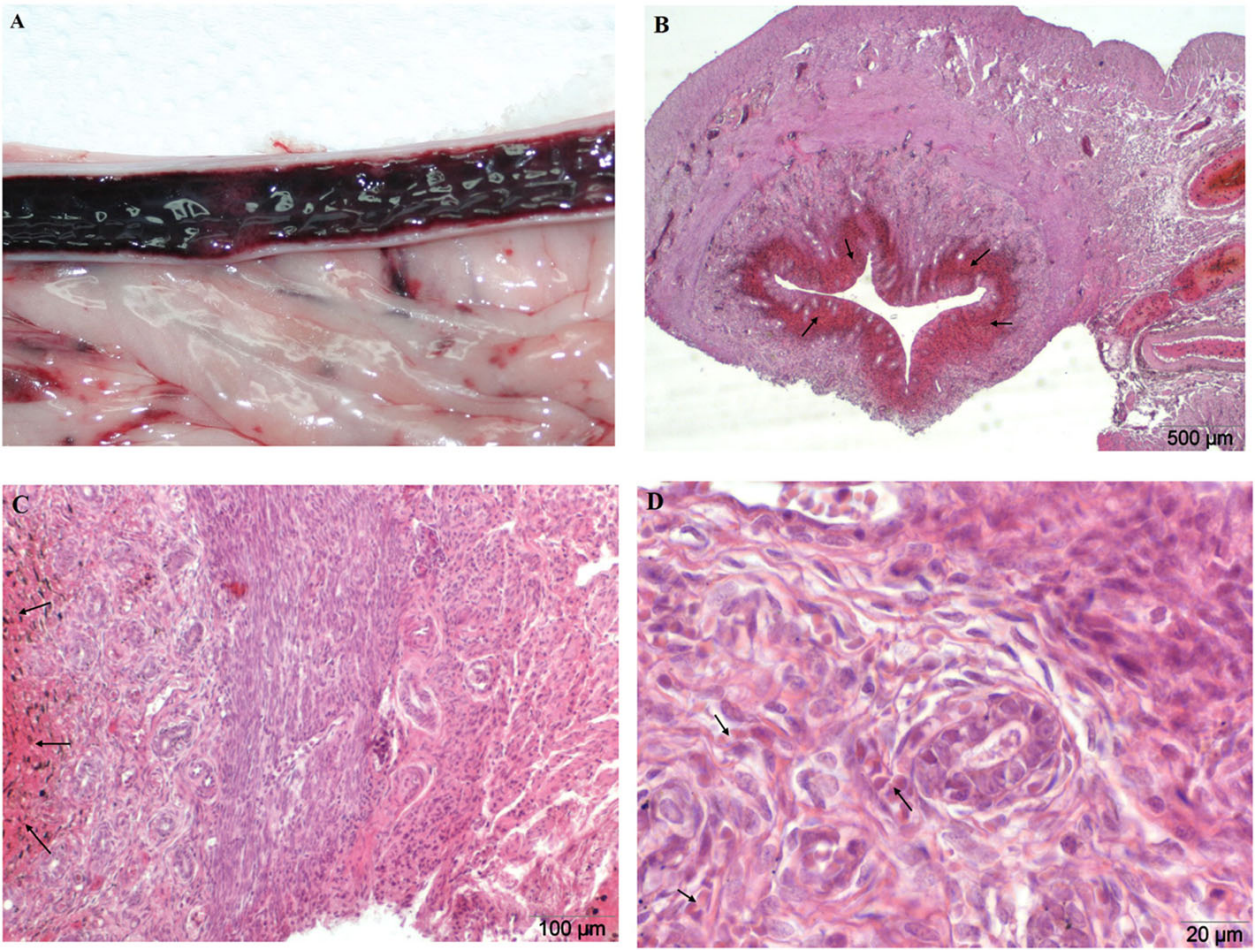
**a-d** Endometrial haemorrhage**.** Uterus from a one-year-old bitch during *anestrus*. A – macroscopic image, longitudinal incision of the uterine horns. Macroscopically visible redness and thickening of the endometrium. **b**, **c**, **d** H&E stained histological images of transverse uterine horn fragment. Microscopically confirmed acute multifocal haemorrhage of endometrium with visible extravascular erythrocytes, a moderate degree of severity (arrows). Magnification: **b** 20x; **c** 100x, **d** 400x

Preliminary macroscopic evaluation of the surface of the uterine mucosa within that group indicated the possibility of the occurrence of pathological changes in the endometrium. The endometrium was clearly reddened and swollen along the uterine horns, which prompted the suspicion of advanced endometritis. However, in the histopathological examination there were no changes observed to indicate an ongoing inflammatory process. Microscopically, the uteri classified into this group showed a moderate (56,7% of cases per group) to high (23,3%) degree of edema subjacent to the luminal epithelium of the endometrium, occurring uniformly within the tested section of the horn. In addition, acute, mostly multifocal endometrial haemorrhage (100%) of mild (20%) to moderate (80%) severity was observed. In rare cases, there were reports of earlier bleeding that underwent a process of resorption (6,7%), as well as small congestion of serous membrane (10%) (Table 1). The appearance of endometrial glands was characteristic of the *anestrus* phase (Fig. 2b, c, d).

### Experimental group II

**Fig. 3:**
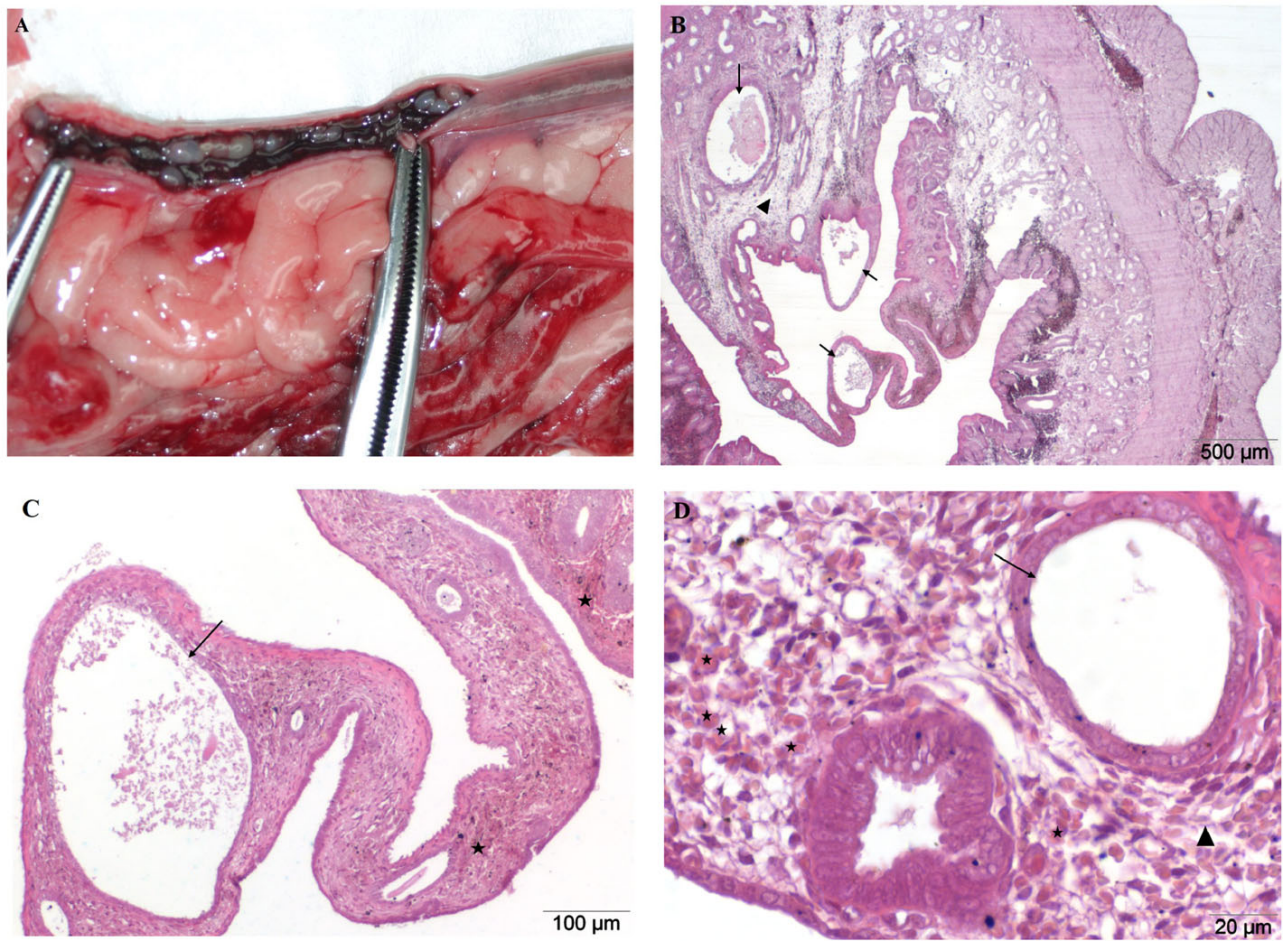
**a-d** Cystic endometrial hyperplasia (CEH). Uterus from an 8 year old bitch. **a** Macroscopic image, longitudinal incision of the uterine horns. Macroscopically observed cysts on the surface of the endometrium. Thickened and reddened mucosa. A small amount of serous-bloody discharge was observed inside the horns. **b**, **c**, **d** H&E stained histological images of transverse uterine horn fragment. Microscopically visible multifocal cystic hyperplasia of glands with a clear flattening of the glandular epithelial cells (arrows) and mild edema of the endometrium (triangle). There are also acute, multifocal endometrial haemorrhage of slight degree (asterisks). Magnification: **b** 20x; **c** 100x, **d** 400x

In the macroscopic evaluation, the CEH covered uterus showed thickened endometrium on its longitudinal section, which was as a result of the presence of numerous cysts on its surface. The degree of histological changes within the endometrial glands in cystic hyperplasia was mild to severe (100%). The microscopic evaluation showed a mild (23,3% cases per group) to moderate (6,7%) endometrial edema. Glands of different sizes, occurring focally and multifocally, were characterized by a great diversity in size and chaotically distributed in the endometrium. Histologically, a significant flattening and progressive atrophy of glandular epithelium was observed. In rare cases a slight (3,35%) to moderate (3,35%) degree of adenomyosis and interstitial fibrosis (6,7%) was identified. There was also an acute, mostly multifocal, mild (10%) to moderately (20%) severe haemorrhage of the endometrium (Fig. 3b, c, d). In most of the analyzed preparations, there was no infiltration of inflammatory cells. In rare cases, a moderate lymphoplasmacytic infiltrate (10%) of the uterine lining was observed (Table 1).

### Experimental group III

**Fig. 4:**
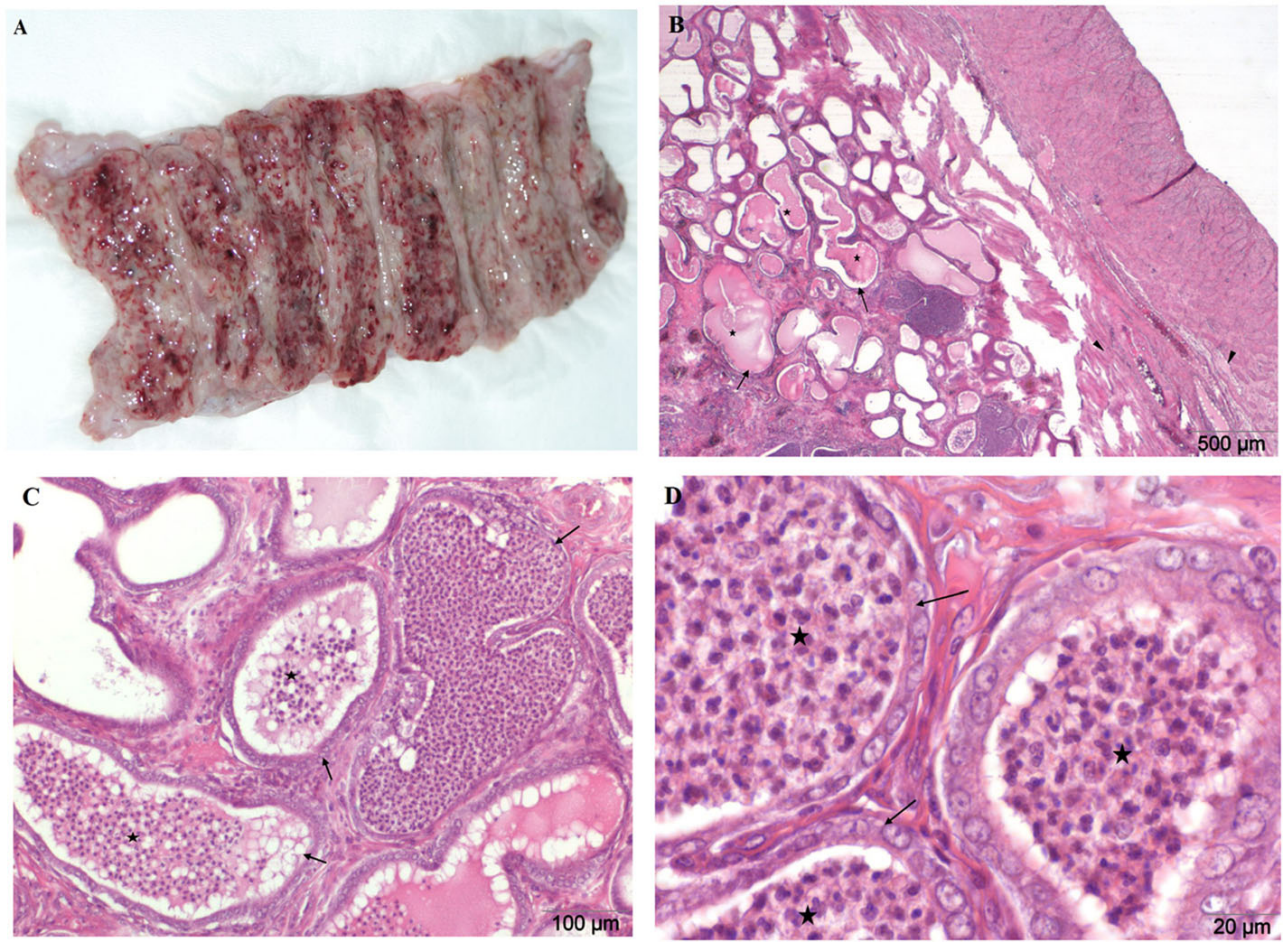
**a**-**d** Pyometra. Uterus from an 8 year old bitch. The uterus increased considerably, horns entirely filled with surging purulent secretions. **a** Macroscopic image, longitudinal incision of the uterine horns. Macroscopically endometrium damaged, presenting significant degenerative changes. A number of differently sized cysts on the surface of the endometrium were observed, most of them already ruptured. **b**, **c**, **d** H&E stained histological images of transverse uterine horn fragment. Chronic, purulent endometritis, with places of lymphoplasmacytic infiltration has been observed. Inflammation of a severe degree occurred in the muscular layer. Multifocal cystic endometrial hyperplasia (arrows) occurred as well as adenomyosis and mild multifocal endometrial fibrosis (triangles). Neutrophils infiltrate the lumen of the endometrial glands (asterisks). Magnification: **b** 20x; **c** 100x, **d** 400x

The uteri obtained from bitches with diagnosed closed (*n* = 22) or open (*n* = 8) type of pyometra were significantly enlarged compared to normal uteri. The increase in volume of the uterine horns was caused by surging purulent secretions in their lumen. Depending on the severity and duration of the inflammatory process and the degree of degenerative changes, discharge took on the color from pale yellow through green to brown. Histologically, in all of the analyzed cases, multifocal cystic endometrial hyperplasia, with varying degrees of severity (100% of cases per group) was also diagnosed. During microscopic examination, neutrophil migration into the lumen of endometrial glands was observed. However, in the endometrial stroma, lymphocytes and plasma cells were found, which demonstrates the chronic inflammatory process (100%). In all uteri there was a considerable degree of inflammation of the endometrium of a purulent, partly lymphoplasmacytic/lymphohistiocytic character, usually diffuse or multifocal, which also comprised of a layer of muscle (Fig. 4b, c, d). Additionally, though in rare cases, there was a moderate to significant degree of, mostly, multifocal fibrosis of the endometrium (16,7%), adenomyosis (13,3%) and endometrial haemorrhage (13,3%) (Table 1).

Statistically significant differences in age of animals were found among control group and other GI, GII, GIII groups; (*P* = 0.004–0.0001). There were no significant differences in the evaluated body weight among the groups. (*P* = 0.76–0.99). The animals were divided into two age groups (< 6 years old and ≥ 6 years old). A statistically significant relationship was detected between bitches age and chronic endometritis in GII and GIII (*P* = 0.0448). The endometritis was often noted in older bitches ≥6 years. Additionally, reddened endometrium in GII and GIII was significantly presented in older bitches ≥6 years (*P* = 0.048). In GI and GII groups, a significant difference in endometrial edema was noted in younger bitches compared to the older ones (*P* = 0.003).

## Discussion

Specific description of cases of proliferative lesions of endometrial glands with different forms of the disease is not easy and often misleading. In addition, the physiology of the reproductive cycle, during which the endometrium of bitches is subject to dynamic reconstruction, does not make the task easier. The research presented recognizes changes in the macroscopic and microscopic image of the endometrium observed in the disease known as CEH-P complex.

De Bosschere et al. [9] points to the importance of research in the pathological diagnosis of the diseases of the uterus in female dogs. According to the authors, a very frequent mistake encountered while making definitive diagnoses of specific diseases of the reproductive system is that vets only clinically assess the state of the organ, but this assessment is not accompanied by detailed research. In women, histopathological examination is the golden rule in the diagnosis of diseases of the uterus [24, 30]. In such cases, an abnormal bleeding or a suspected infertility are the indicators for advanced diagnostics. It is a routine test in the case of a chronic inflammation of the lining of the uterus, and the identification of plasma cells in the endometrial stroma provides the basis for a diagnosis [24].

Lesions involving the uterus were classified according to the severity of CEH-P. In the macroscopic and microscopic assessment of the uterus of a bitch with a significant advancement of the disease, the changes typical of classical pyometra were noted. The significant enlargement of the organ, congestion and thickening of the endometrium as well as residual purulent exudates in the lumen of the uterus were observed. Microscopic examination of the sections of the uterine horns showed structural changes typical of CEH, including proliferation, together with the presence of cysts on the surface of the endometrium (Fig. 4b, c). In addition, the proliferation of the endometrial epithelial lining was observed as well as the transformation of the endometrial glandular epithelium (secretory phase).

The results presented in this study as well as the authors cited for all cases of the analyzed uteri, describe a considerable degree of endometritis, mainly purulent. Inflammatory infiltration, primarily of mononuclear cells, demonstrating a chronic (100% of cases per group), inflammatory process was identified within the endometrial stroma. However, on the mucosal surface and in the lumen of the uterine glands a plurality of neutrophils was observed (Fig. 4c). In addition, though rare, there were cases of moderate to significant degrees of, mostly, multifocal fibrosis (16,7%) and endometrial haemorrhage (13,3%).

A picture typical of CEH-P was also observed by Groppetti et al. [12]. Apart from cystic and inflammatory processes, instances of CEH-P were accompanied by swelling of the endometrial stroma, infiltration of inflammatory cells and mucosal haemorrhage, which has also been described in this research.

The research findings by Younis et al. [17] also confirm the existing changes in the macroscopic and microscopic image observed in this study. In the group of uteri affected by CEH-P, the authors chose three characteristic histological changes typical of the disease. The first one included the purulent uterine content, which in some cases occupied the deeper layers of the uterus. Another one was referred to the changes associated with pathological enlargement and proliferation of endometrial glands (CEH). The last, third group of changes included - progesterone dependent - proliferation of glandular epithelium, showing vacuolization of the cytoplasm and a single pyknotic nucleus. To a lesser or greater extent, the observed histopathological changes were also recorded by Bigliardi et al. [4].

An analysis of the age structure of bitches with particular uterine diseases in our study confirms the development of pyometra occurring more often in older females, aged about 9.3 years, while CEH affects bitches over 6 years of age (*p* < 0,001). Although both diseases are distinct conditions included in the complex pathology of the uterine diseases of bitches, the importance of CEH in the development of pyometra is unquestionable [5, 6, 9, 31].

Relating our own results to the works cited, it should be noted that among the analyzed cases of pyometra, there were also advanced, multifocal cystic hyperplasia of endometrial glands in all of them (100% of cases, Fig. 4b, c). The histological picture refers to class VI of the classification of uterine diseases described by de Bosschere et al. [9] as pyometra (hyperplastic) with severe inflammatory response, numerous cysts, an overstated relation of endometrium to myometrium as well as moderate proliferation of fibroblasts.

Although cystic endometrial hyperplasia glands (CEH) are encountered in both bitches and queens, the disease more often affects the bitches. This is associated with a longer period of impact of progesterone on the endometrium during the *diestrus* [1, 32, 33]. In the literature, many studies describing abnormal endometrial hyperplasia in relation to diseases like CEH, also mark the likelihood of its progress to life-threatening stages [14, 25, 31–37]. Cysts developing in the endometrium classified as CEH differ significantly in terms of number, size, distribution and histomorphology [35]. Groppetti et al. [12], in their microscopic evaluation of CEH, observed a clear hyperplasticity and thickening of the tissue with endometrial stromal edema and a significant extension and branching of glands. The diameter of the cysts identified by them amounted to a few millimeters, and the interior was filled with clear secretions of mucous character. Both the superficial and glandular epithelium of the endometrium formed high and irregular stratified columnar cells.

Compatibility of the described results was also achieved in our research; microscopic analysis of histologic preparations of uteri affected by CEH revealed a wide range of observed changes ranging from mild (13,3% cases per group) to advanced (86,7%). The histologic evaluation was dominated by an increase in glands of the endometrium, with the occurrence of both focal and multifocal kinds. In addition, we observed a significant flattening and progressive atrophy of the glandular epithelium as well as the often acute, multifocal haemorrhage of the endometrium (30%), which has not been included in the analyses of the previously cited authors (Fig. 3b, c). Using the classification of uterine diseases used by De Bosschere et al. [9], the presented conversion, which does not include an inflammatory response, is eligible for the third group - described as a severe form of CEH with numerous large cysts and increased relation of endometrium to myometrium.

During the initial evaluation of the research material, the uteri of the bitches from the experimental group I (GI) have been characterized as falling within the range of moderate inflammation. Such a finding is possible thanks to the macroscopic evaluation of the endometrium of clinically heathy bitches in the *anestrus* phase. A clearly reddened and thickened endometrium, in some cases, with a small amount of serous-bloody discharge, suggested the classic picture of endometritis (Fig. 2a). Meanwhile, microscopic research has not confirmed any inflammation of the lining of the uterus, and, what is more, there was no presence of any cells that provide for ongoing inflammatory process within this organ, but only multifocal endometrial haemorrhage (100% of cases per group) with mild (20%) to moderate (80%) severity (Fig. 2b, c). In some cases uteri classified into this group showed a moderate (56,6%) to high (23,3%) degree of endometrial edema, occurring uniformly within the tested section of the horn. The results obtained by our studies confirm the need to perform detailed analyses in the differentiation of conditions of the uterus in bitches due to the lack of consistency of macroscopic assessment with histological analysis. The presented results, however, require the continuation of studies and more detailed biological analysis, due to the lack of data clarifying the described condition.

As indicated by published literature, the clinical condition of the patient does not constitute grounds for the exclusion of uterine diseases [1, 2, 9]. The obtained studies reported that the majority of bitches, which were classified as clinically healthy, were found to have uterine or ovary disorders in varying degrees of progression. In De Bosschere et al. [9] this problem concerned 20 out of 26 patients identified as having hyperplastic changes of the endometrium from mild (8) to heavy CEH (9) - both in the *diestrus* and *anestrus* phase. Additionally, in some cases, stromal edema, endometrial haemorrhage and local endometrial bleeding was diagnosed. These results are consistent with our own observations, where the majority of cases of haemorrhage of the endometrium and hyperplasia of glands, occurred in clinically healthy bitches who had undergone ovariohysterectomy for the purpose of reducing the risk of later development of uterine diseases and depression of fertility.

## Conclusions

The results present descriptive studies and indicate significant changes in the macroscopic image and microscopic structure of the endometrium, observed in the analyzed uterine diseases. The early reproduction disorders in bitches are, in most cases, not confirmed by clinical signs in the examined animals. This is important with respect to the reduced fertility of unknown grounds in bitches that are valuable for breeding. The research recognizes significant changes in the microscopic structure of the endometrium observed in diseases of the uterus, forming part of a complex disease known as CEH-P. Moreover, they indicate the importance of research in the pathological diagnosis of uterine diseases in bitches and show a possible lack of consistency in the macroscopic assessment and histological analysis.

Despite the difficulties and risk of endometrial biopsy in bitches, the obtained results of macroscopic evaluation and histological analysis may be useful tools in the evaluation of the collected tissue. In addition, the presented classification of uterine diseases, which was created after detailed histological analyses, may prove to be useful for scientists in the field of basic research.

## Methods

### Animals

Material for the study consisted of the uteri of 120 female dogs, aged 1 to 16 years, obtained during routine ovariohysterectomies (OVH), performed at the request of their owners. All animals underwent clinical assessment. Ovariohysterectomy was performed at specified times depending on the stage of the reproductive cycle.

Immediately after the surgery, the uterus and ovaries were transported to the laboratory at room temperature. The animals from which the material was collected had not previously received hormone therapy or given birth. More details about their status are presented in Table 2. After the OVH procedure, the researchers conducted a gross examination for the detection changes in the collected uteri and ovaries. Only uterine material which was not accompanied by ovarian pathologies (manifested in macroscopic evaluation) was collected for further analysis. In the *anestrus* phase (control, GI group) ovaries were typically small and inactive. Mostly normal ovaries with mature *corpus luteum* were observed during the *diestrus* phase (GII and GIII groups). Pathological changes in the ovaries, such as cysts on the ovarian capsule or cortex, were found only in 4 bitches with CEH and 6 with pyometra, and material from these animals was excluded from the study.

**Table 2:** Details of the bitches used in the research

Number of bitches	Control group	Group I Endometrial Haemorrhage	Group II CEH	Group III ***Pyometra***
	*n* = 30	*n* = 30	*n* = 30	*n* = 30
				Open type = 8
				Close type = 22
**Age (years)**				
Mean age of bitches (years) ± SD	2,2 ± 1,5^*^	4 ± 2,4^*^	6,4 ± 2^*^	9,3 ± 2^*^
max	8	14	13	16
min	1	1	3	2
**Body weight (kg)**				
Mean weight of bitches (kg) ± SD	17.1 ± 12,3	18.7 ± 11,3	20.2 ± 8,9	19.5 ± 14,8
max	54	42	40	53
min	2.3	5	6	3
**Breed**				
crossbreed	22	15	12	14
in breed type	8	15	18	16

^*^ significant differences among control group and GI, GII, GIII, *P* value < 0,01

The phase of the sexual cycle was determined on the basis of cytology smears taken from the vagina. The control group comprised females during the *anestrus* phase; group I included *anestrus*, groups II and III consisted of females during the *diestrus* phase*.*

The uterine was examined in order to look for the presence of any kind of secretion or macroscopic abnormalities. Macroscopic observation, after a longitudinal incision of the uterine horns, allowed the preliminary classification of the animals into four study groups: a control group and group I – with endometrial haemorrhage, group II- bitches with CEH and group III with pyometra.

### Clinical evaluation of animals

The animals of the control, GI and GII group were in an optimum body condition and did not show any clinical symptoms characteristic for diseases of the reproductive system. Before OVH intervention, a hematologic evaluation was performed in each bitch. In the control, GI or GII study groups, all dogs that underwent the surgical procedure presented normal morphological and biochemical parameters in blood. Only the macroscopic evaluation of the obtained material, after the OVH procedure, allowed for the preliminary division of the examined individuals into the control group, GI and GII.

Bitches in group GIII had clinical symptoms associated with pyometra in diverse intensity. Animals diagnosed with pyometra presented different stages of typical symptoms, such as purulent or purulent–hemorrhagic discharge, lethargy, vomiting, polydipsia, polyuria, dehydration, anorexia, abdominal enlargement, temperature disturbances and changes in the color of mucous membrane. Hematological and blood biochemistry changes were also observed. In the WBC count a differential left shift has been observed as well as leukocytosis with neutrophilia. Moreover, normocytic or normochromic anemia and thrombocytopenia has been noted. In biochemistry analysis of the blood, C–reactive protein, alkaline phosphatase, bilirubin, cholesterol, creatinine, blood urea nitrogen and electrolytes were increased.

### Macroscopic evaluation and preliminary classification of research material with regard to listed pathological changes

The morphological diagnosis and histologic description of samples were noted. A preliminary assessment of lesion severity was recorded and confirmed by the histopathology report. Macroscopic observation, after longitudinal incision of the uterine horns, of characteristics such as degree of redness, thickening of the endometrium, cysts on its surface or presence and color of discharge allowed us to classify the uteri into four study groups. These preliminary classifications were then verified by histological analysis.

The control group (C, *n* = 30), consisted of uteri from clinically healthy bitches with no inflammatory alterations or uterine cysts. The macroscopic estimate is presented in Fig. 1a. Uteri with pathological changes in the mucous membranes were divided into three groups. The experimental group I (GI, *n* = 30) consisted of uteri which were initially evaluated as being in the state of inflammation. They were strongly congested and the mucous membrane of the uterus was thickened (Fig. 2a). Group II (GII, n = 30) consisted of uteri affected by cystic endometrial hyperplasia (CEH), and in such cases numerous multifocal cysts on the surface of the endometrium, filled with serous exudates, were found. Serosanguinous discharge in the lumen of the horns has been reported in rare cases (Fig. 3a). The last experimental group consisted of uteri with accumulation of purulent secretions, residual in the lumen of their horns (GIII, n = 30). These uteri were significantly enlarged, mainly presenting pyometra of a closed type (Fig. 4a).

The initial distribution of uteri in the experimental groups was based on the classification suggested by De Bosschere (2001).

### Histological staining and visualization of microscopic preparations

Obtained fragments of the sections of the uterine horns, of approximately 2–3 cm, were fixed in buffered 10% formalin, and then dehydrated in a series of increasing concentrations of ethanol and placed in xylene with the aim of exposing the tissues and removing the alcohol from them. Tissues prepared in this way were then embedded inside a block of paraffin. The paraffin blocks were cut into slices using a rotary microtome. Paraffin was removed using xylene, and the preparations were then hydrated in a series of decreasing concentrations of alcohol after which they were finally placed in water. Staining of the preparations was performed on the basis of the staining method of Böck [38] using hematoxylin and eosin. After staining and dehydration of the preparations in a series of increasing concentrations of alcohol, they were secured by gluing them to cover slips using a lotion (Leica CV MOUNT). The preparations were evaluated under a light microscope (Olympus WX41). Pictures were made using the Image Analysis Software “analySIS FIVE”. The obtained results were confirmed by a certified pathologist.

### Statistical analysis

Data were analyzed using the commercial statistical software GraphPad Prism 7.0. Animal age and weight statistics are expressed as mean ± standard deviation (SD). Normality of distribution was verified using the Shapiro–Wilk test. The statistical analysis (age and weight) between the examined groups C, GI, GII, GIII was performed using ANOVA followed by the Tukey post hoc test.

The number of bitches (from each test group) showing the current uterine pathologies, were compared to the age. The animals were divided into two parts for statistical analysis age groups (< 6 years old; ≥6 years old). Comparisons of individual variables between groups were made using the χ2 independence test. Statistically significant differences were taken into account when *P* < 0.05.

## Data Availability

The datasets used during the current study are available from the corresponding author on reasonable request.

## Abbreviations

CEH: Cystic endometrial hyperplasia
CEH-P: Cystic endometrial hyperplasia - pyometra complex
CH: Complex hyperplasia
H&E: Hematoxylin and eosin
OVH: Ovariohysterectomy
WBC: White blood cells
